# Supplementary material for: ESCRT-III is necessary for the integrity of the nuclear envelope in micronuclei but is aberrant at ruptured micronuclear envelopes generating damage
Source: Oncogenesis. 2019 Apr 15;8(5):29. doi: 10.1038/s41389-019-0136-0 (PMC6465242; doi:10.1038/s41389-019-0136-0)
Supplement: Supplementary file 1 — Supplementary figure and legends. [file 41389_2019_136_MOESM1_ESM.pdf]

Supplementary figure and legends

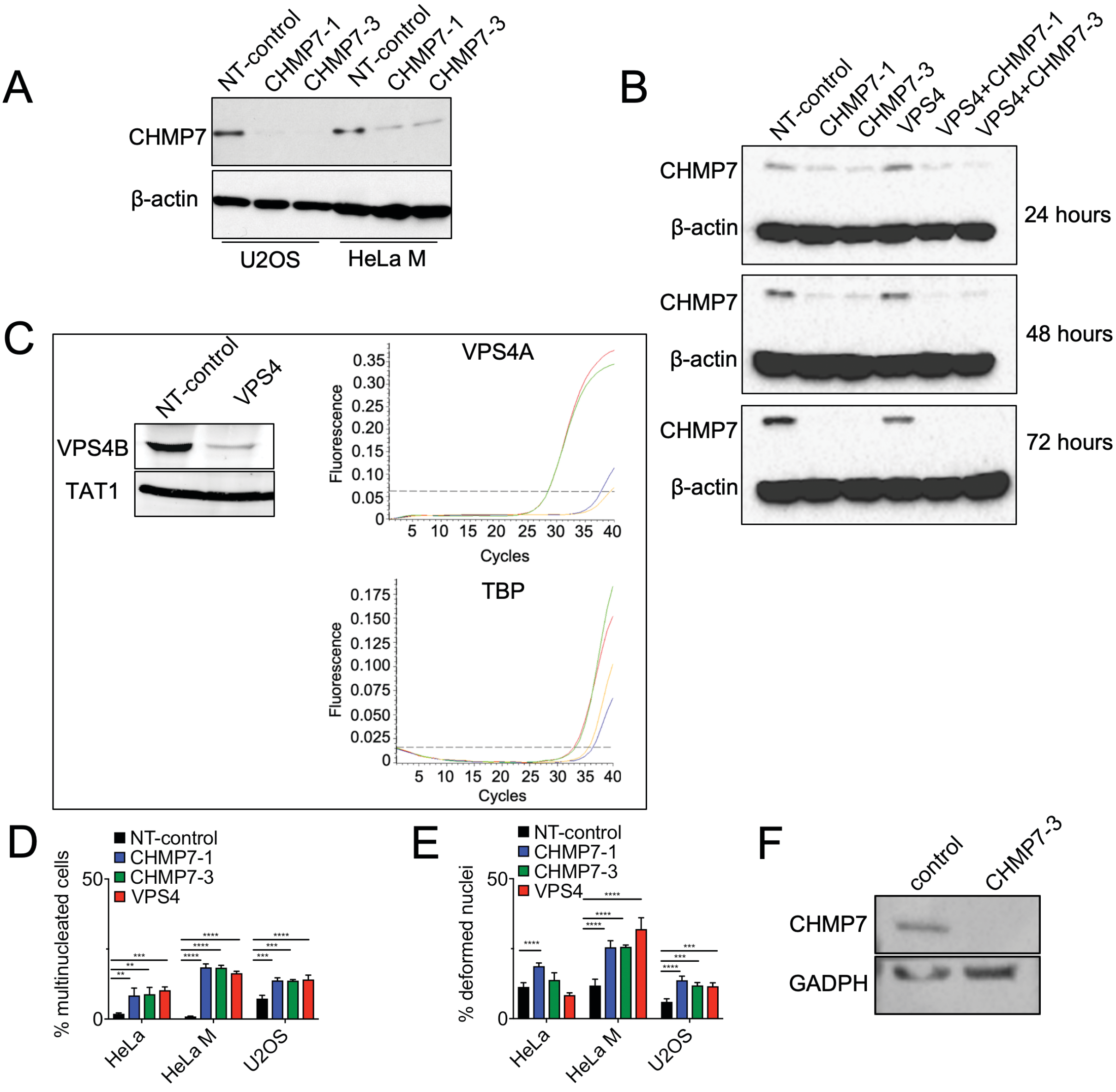

Supplementary Figure 1. RNAi knockdown efficiency and nuclear effects. (A) Western blot showing knockdown of CHMP7 in U2OS cells by the two CHMP7 sRNAi sequences used in this study. U2OS cells were treated with 10 nM sRNAi for 48 hours. The samples were blotted using the mouse-derived anti-CHMP7 antibody. (B) Levels of CHMP7 protein in sRNAi knockdown of CHMP7 and VPS4 individually

and in combination in HeLa cells at 24, 48 and 72 hours post-transfection. The samples were blotted with the mouse-derived anti-CHMP7 antibody. (C) HeLa cells were treated with 5 nM sRNAi of each VPS4A and B oligos for 48 hours. Left: Western blot showing knockdown of VPS4B. Right: amplification curves from qPCR showing depletion of VPS4A in comparison to the housekeeping gene TBP as an internal standard (VPS4A primer top, TBP primer bottom; 2 independent runs. Red/green lines are for control KD, orange/blue lines are for VPS4A/B KD). The VPS4A KD efficiency is ~98%. (D) Quantification of multinucleated cells and deformed nuclei phenotypes (E) generated upon treatment of Hela cells with the indicated sRNAi (a minimum of 500 cells were scored per condition). Results were analyzed using a two-way ANOVA with Dunnett's post hoc test. Averages and SEM (N=3) are shown. (F) Western blot showing knockdown of CHMP7 in h-TERT fibroblasts after treatment with 20 nM CHMP7-3 sRNAi for 48 hours. Control cells were transfected with sRNAi GLi2 20 nM. In all panels, NT-control is non-targeting siRNA. VPS4 represent co-depletion of VPS4A and B.

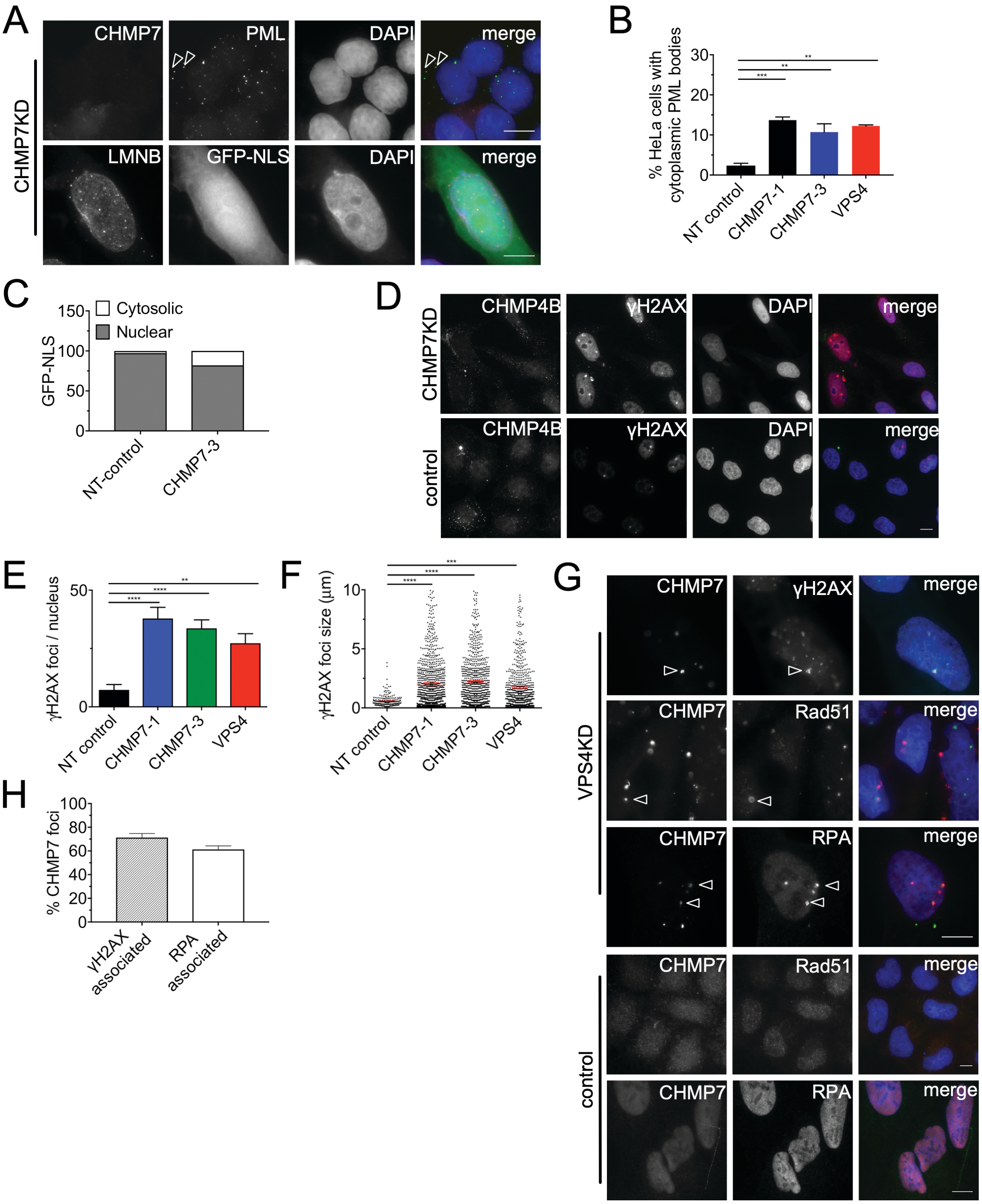

Supplementary Figure 2. ESCRT-III impairment predispose cells to DNA damage. (A) Cytoplasmic PML bodies in HeLa cells (arrowheads indicating cytoplasmic PML) and GFP-NLS localization in h-TERT immortalised human fibroblast after treatment with CHMP7 sRNAi for 48 hours. (B) Quantification of HeLa cells with cytoplasmic

PML bodies across NT control, CHMP7 and VPS4 sRNAi transfection (minimum of 550 cells scored per condition). Results were analysed using a one-way ANOVA with Dunnett's post hoc test. (C) Quantification of GFP-NLS retention judged visually by the gain in cytoplasmic signal in h-TERT fibroblast treated for 24 hours with the indicated sRNAi and then transfected with GFP-NLS and fixed at 48 hours post transfection (between 64 and 148 cells scored). Averages of N=2 biological repeats are shown. (D) Nuclear γH2AX foci in HeLa cells, after treatment with control or CHMP7 sRNAi for 48 hours. (E) Quantification of the number of γH2AX foci in the nucleus in Hela cells treated with the indicated sRNAi for 48 hours (minimum of 30 cells per conditions). (F) Diameter of γH2AX foci (NT-control – 218 foci; CHMP7-1 – 1587 foci; CHMP7-3 – 1076 foci; VPS4 – 1076 foci) in HeLa cells treated with the indicated sRNAi for 48 hours. Results were analysed using a one-way ANOVA with Dunnett's post hoc test. (G) CHMP7 foci at the nuclear periphery co-occur with markers of DNA damage and repair. HelaM cells transfected with control or VPS4 sRNAi for 48 hours, co-stained to show CHMP7 and either γH2AX, Rad51 or ssDNA detected using RPA34 (bottom panel, U2OS cells). (H) CHMP7 foci were scored for co-occurrence with γH2AX or RPA in HeLa cells transfected with VPS4 sRNAi for 48 hours (a minimum of 300 foci were scored for each condition). Averages and standard error of three independent repeats are shown, except for (C). Scale bars represent 10 μm.

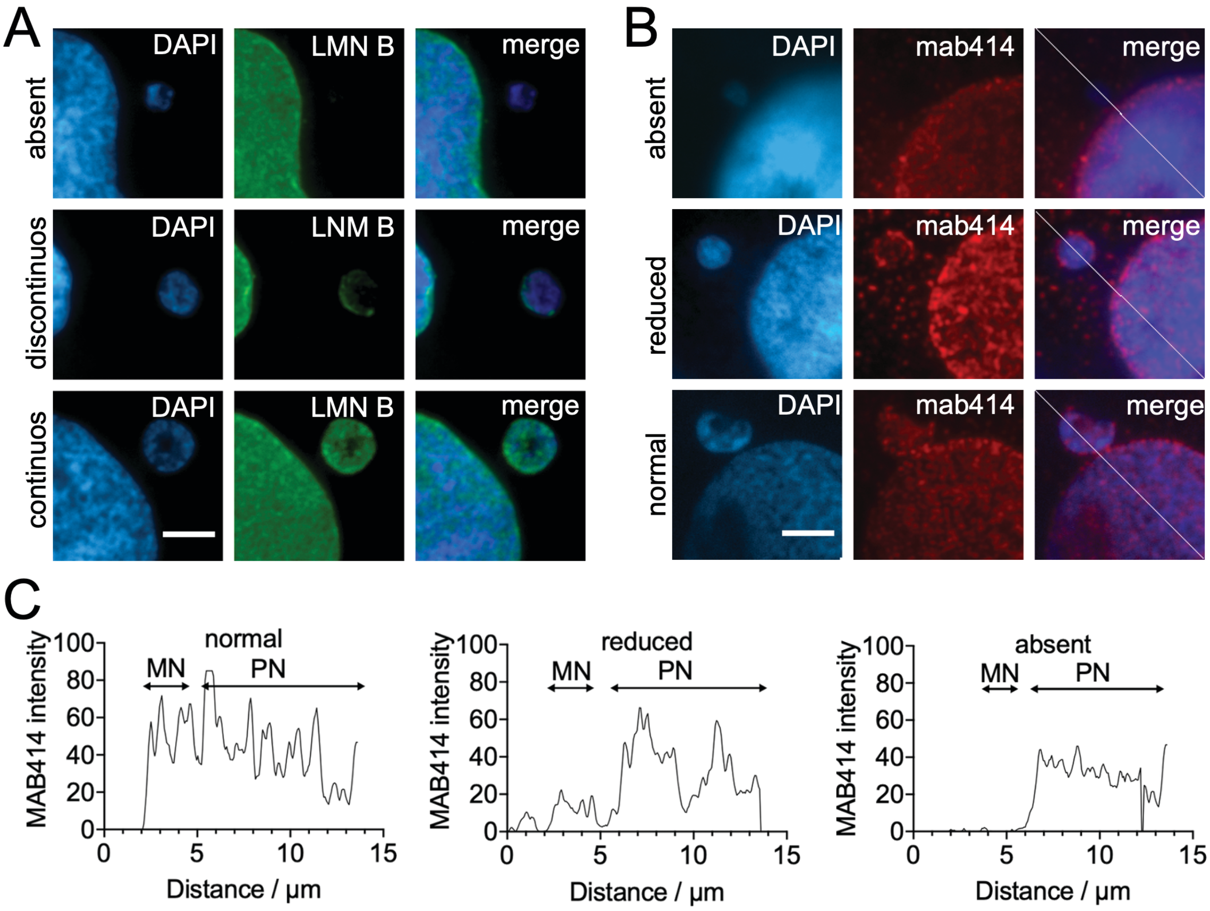

Supplementary Figure 3. (A) Categories of micronuclei where the nuclear lamina is intact (continuous), broken (discontinuous) or entirely absent. Scale bar 3 µm. (B) Categories of micronuclei where the NPCs density by visual inspection is present at normal levels, at reduced levels or are entirely absent. Scale bar represents 3 µm. (C) Relative pixel intensity of the MAB414 signal at the micronucleus (MN) and primary nucleus (PN).

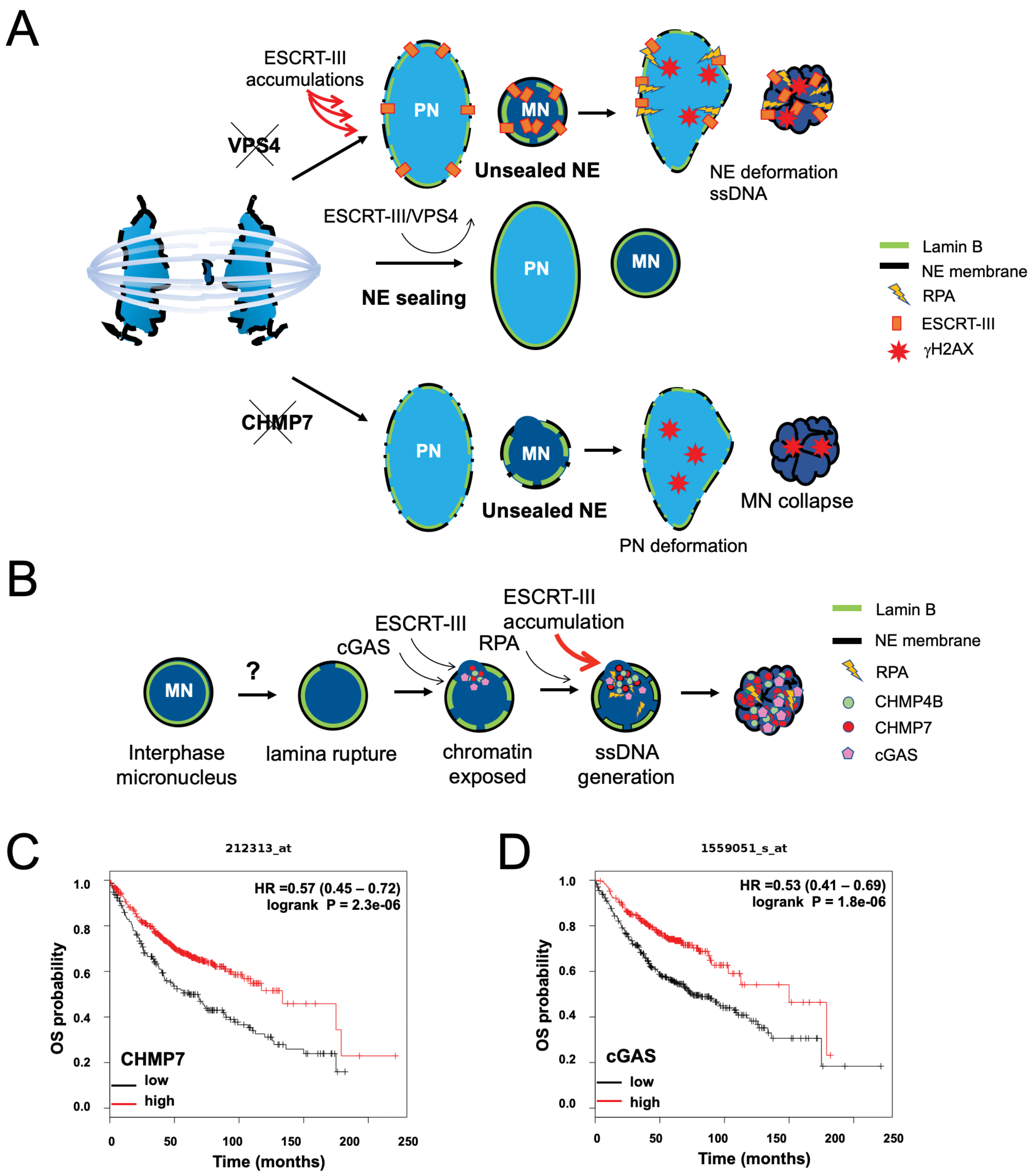

Supplementary Figure 4. (A) Perturbation of ESCRT-III activity increases the population of micronuclei with disrupted nuclear envelope (NE). ESCRT-III and VPS4 recruitment and activity at mitotic exit proceeds normally at the primary nucleus (PN) and micronucleus (MN). This pathway of ESCRT-III recruitment is VPS4-dependent. In the absence of VPS4, ESCRT-III activity is unregulated, resulting in

excess ESCRT-III recruitment with consequent membrane deformation and DNA damage. In the absence of CHMP7, ESCRT-III is not recruited at NE gaps, resulting in the generation of PNs and MNs with weak and leaky NE. (B) Ruptured micronuclei (MN) in interphase cells contain accumulated ESCRT-III. These micronuclei develop collapsed envelopes, accumulate single-stranded DNA and expose genomic material to the cytosol, with the build-up of ESCRT-III exacerbating damage. This pathway of ESCRT-III accumulation is VPS4-independent. (C-D) Overall survival curves of lung adenocarcinoma analyzed for CHMP7 (C) and cGAS (D) expression using KM plotter (www.kmplot.com)[1] using an auto-select cutoff. For CHMP7: 720 patients; probe=212313_at; logrank P=2.3e-6 (squamous cell carcinoma not significant). For cGAS: 720 patients; probe=1559051; logrank P=1.8e-6 (squamous cell carcinoma not significant).

Additional references

1 Györffy B, Lanczky A, Eklund AC, Denkert C, Budczies J, Li Q *et al.* An online survival analysis tool to rapidly assess the effect of 22,277 genes on breast cancer prognosis using microarray data of 1,809 patients. *Breast Cancer Res Treat* 2010; **123**: 725–731.

Supplementary tables

Supplementary table 1

sRNAi target	sRNAi oligonucleotide sequences
**NT-control**	ON-TARGETplus non-targeting control sRNAi (#1; Dharmacon)
**CHMP7-1***	GGGAGAAGAUUGUGAAGUU
**CHMP7-3**	GGAGGUGUAUCGUCUGUAU
**VPS4A oligo 5****	CCACAAACAUCCCAUGGGU
**VPS4A oligo 6****	CCGAGAAGCUGAAGGAUUA
**VPS4B oligo 5****	GGGCAAAGUGUACAGAAUA
**VPS4B oligo 6****	CGAUAGAUCUGGCUAGCAA

* from Morita et al PNAS 2010

** from Stefani et al Current Biology 2011

Supplementary table 2

Primary antibodies					
Antibody (α-Hu)	Host Species	Source	Code	Dilution	
				WB	IF
**α- β-actin**	mouse	Proteintech	66009-1-IG	1:10000	1:100
**α-CHMP4B**	rabbit	Proteintech	13683-1-AP	1:1000	1:200
**α-CHMP7**	mouse	Santa Cruz	SC-271805	1:1000	1:250
**α-CHMP7**	rabbit	Proteintech	16424-1-AP	1:1000	1:200
**α-cGAS**	rabbit	Cell Signalling Tech	15102	X	1:200
**α-CREST**	human	Antibodies Inc	15-234-0001	X	1:50
**α-GADPH**	mouse	Santa Cruz	SC-32233	1:1000	X
**α-γH2AX**	mouse	Abcam	ab11174	X	1:1000
**α-γH2AX**	rabbit	Cell Signalling Tech	9718	X	1:400
**α-Lamin A/C**	goat	Santa Cruz	SC-6215	X	1:500
**α-Lamin B1**	rabbit	Proteintech	12987-1-AP	X	1:300
**α-LAP2**	rabbit	Proteintech	14651-1-AP	X	1:300
**α-PARP1**	mouse	Calbiochem	AM30	X	1:500
**α-PDI**	rabbit	Cell Signalling Tech	2446	X	1:500
**α-PML**	rabbit	Abcam	Ab53773	X	1:200
**α-RAD51**	rabbit	Santa Cruz	SC-8349	X	1:100
**α-RPA32**	rabbit	Bethyl Labs	A300-245A	X	1:1000
**α-RPA34-20**	mouse	Calbiochem	NA19L	X	1:200
**α-RPA70**	mouse	Santa Cruz	sc-48425-S	X	1:200
**α-TAT1**	mouse	*	*	1:100	X
**α-VPS4B**	rabbit	Proteintech	17673-1-AP	1:1000	X

* kind gift from Keith Gull, University of Oxford 1:100 of a hybridoma supernatant
